# Pioglitazone suppresses excessive follicular development in murine preantral follicles

**DOI:** 10.1186/s13048-019-0556-7

**Published:** 2019-08-31

**Authors:** Sachiko Nagao, Tsuyoshi Baba, Yuya Fujibe, Sayaka Adachi, Keiko Ikeda, Miyuki Morishita, Yoshika Kuno, Hiroyuki Honnma, Toshiaki Endo, Tamotsu Kiya, Tsuyoshi Saito

**Affiliations:** 1 Present address: Department of Obstetrics and Gynecology, Sapporo Medical University, South 1 West 16, Sapporo, Hokkaido 060-8543 Japan; 2 Sapporo ART Clinic, 1-4 North 7 West 4, Sapporo, Hokkaido 060-0807 Japan; 3 Ena Ladies Clinic, Hanakawa South 9-1-86-2, Ishikari, Hokkaido 061-3209 Japan

**Keywords:** Pioglitazone, Androgen, Follicle-stimulating hormone, Follicular development, Growth factors

## Abstract

Polycystic ovary syndrome (PCOS) is an endocrine disease that is common in women in their reproductive period. Patients with this disease suffer from anovulation and hyperandrogenism. Ovulation induction with exogenous gonadotropin often causes ovarian hyperstimulation syndrome because many small antral follicles pause in their growth. Treatment with insulin sensitizers is reportedly effective for both anovulation associated with PCOS, and suppression of excessive follicular growth; however, the underlying mechanism of action remains unknown. Although pioglitazone is known as an insulin sensitizer, it also has a potent modulator of cell growth and apoptosis irrespective of insulin resistance. To clarify the effect of pioglitazone on follicular growth, we performed in vitro culture of murine preantral follicles. Secondary follicles (100-160 μm in diameter) isolated from 6-week-old ICR mice were individually cultured for 13 days. Culture conditions were as follows: 1) follicle-stimulating hormone (FSH; 33 mIU/mL; control), 2) FSH plus dihydrotestosterone (DHT; 500 ng/mL), 3) FSH plus pioglitazone (5 ng/mL), and 4) FSH plus DHT/pioglitazone. Survival rate and follicle diameter were evaluated, and concentrations of estradiol (E2) and vascular endothelial growth factor (VEGF) in culture media were measured. mRNA expression of various growth-promoting factors and *Vegf* within follicles were also assessed. Although no significant differences were observed with regard to survival rate, follicle diameters on day 13 were significantly different.

Compared with the control group, the DHT group showed enhanced growth, while groups administered pioglitazone showed stagnation of the accelerated growth induced by DHT. Although DHT treatment enhanced the expression of bone morphogenetic protein 2 (*Bmp2*) mRNA, pioglitazone exposure suppressed induction of *Bmp2* mRNA by DHT. *Vegf* mRNA and protein expression were also significantly reduced when pioglitazone was added to culture media containing DHT.

Administration of pioglitazone negatively affected follicular growth and VEGF levels, which may suppress excessive follicular growth and prevent ovarian hyperstimulation syndrome.

## Background

Polycystic ovary syndrome (PCOS) is an endocrine disease that is common among women in their reproductive period. Characteristics of PCOS include anovulation due to cessation of follicular growth, clinical or biochemical hyperandrogenism, and polycystic ovary morphology (PCOM) [1, 2]. In addition, many women with PCOS exhibit insulin resistance. Ovarian hyperstimulation syndrome (OHSS) frequently occurs when ovulation-inducing agents are administered to PCOS patients, which interferes with treatment. In severe cases in which diagnosis and treatment are not properly performed, irreversible and severe disability or death can occur. Although insulin sensitizers are reportedly effective against anovulation associated with PCOS or for suppression of excessive follicular growth [3, 4], the underlying mechanism of action remains unknown. However, the complexity of follicular growth, which involves contributions from numerous growth factors and pathways, makes it difficult to define the cellular mechanism of insulin sensitizer.

In general, the effects of insulin resistance and subsequent hyperinsulinemia on ovarian function are thought to occur as follows: insulin promotes androgen secretion through luteinizing hormone (LH) stimulation to increase the number of initial antral follicles by increasing free androgen via decreased sex hormone-binding globulin (SHBG), which leads to follicular atresia as a result of continuously high androgen levels [5]. Intriguingly, exposure to high levels of androgen during the embryonic stage of development may bring about insulin resistance [6, 7], indicating a relationship between insulin resistance and elevated androgen levels.

Many studies have reported that insulin sensitizers improve insulin resistance and reduce high androgen levels, subsequently ameliorating ovulation disorders in women with PCOS [8–10]; in particular, use of metformin has become common [11, 12]. In the present study, we utilized the thiazolidinedione derivative pioglitazone, an insulin sensitizer approved for clinical use in Japan. Thiazolidinedione derivatives stimulate insulin signal transmission downstream of insulin receptor binding without stimulating insulin secretion, thereby improving insulin resistance. In addition, increased adiponectin secretion may mitigate the disease by decreasing free androgens through SHBG [13, 14].

Many previous studies have investigated the effects of androgen administration on ovarian function using animal models of PCOS. However, experimental animals (particularly rodents) do not completely simulate PCOS when androgens are administered. One reason for this is that the effect of high androgen levels on ovaries is thought to be modulated through actions of the hypothalamus and pituitary gland during in vivo experiments. We previously reported that administration of dihydrotestosterone (DHT) to murine secondary follicles in a low follicle-stimulating hormone (FSH) environment increased FSH receptor expression and promoted follicular growth by enhancing FSH action [15]. Based on these results, we prepared secondary follicle cultures in a low FSH/DHT environment as a PCOS model, and utilized this model to study the effects of insulin sensitizers on PCOS.

## Methods

Ovarian follicles consist of oocytes, granulosa cells, and theca cells, which interact with each other to develop competent (ability to be fertilized) oocytes. Follicular growth and steroid production are regulated by various factors, such as pituitary gonadotropins, steroid hormones, and local growth factors. Thus, to investigate the effects of androgens and insulin sensitizers on follicular growth in the absence of effects of the hypothalamus and pituitary gland, follicles were isolated and subjected to in vitro culture individually.

### Animals and cell culture

Female ICR mice were obtained from Sankyo Labo Service Corporation (Sapporo, Japan). Mice were handled according to guidelines provided by Sapporo Medical University and the Scientists Center for Animal Welfare. Protocols were approved by Sapporo Medical University Institutional Animal Care and Use Committee. Six-week-old female ICR mice (*n* = 4) were euthanized via intraperitoneal injection of pentobarbital (120 mg/kg). Ovaries were removed and secondary follicles (100–160 μm in diameter) were mechanically isolated using 30-gauge needles under an inverted microscope. Follicles with an intact basement membrane, clear granulosa cell layers and oocytes, and centrally located round oocytes were selected for the study. Each follicle was placed individually into wells of a 96-well multiple cell-repellent surface plate (Corning, Corning, NY) containing 200 μL of Alpha Minimum Essential Medium (Thermo Fisher Scientific, Waltham, MA) supplemented with 5% fetal bovine serum (Corning), 10 μg/mL insulin, 5.5 μg/mL transferrin, 6.7 ng/mL sodium selenite, 200 IU/mL penicillin (Thermo Fisher Scientific), and 33 mIU/mL FSH (Sigma-Aldrich, St. Louis, MO). A previous study showed that the minimal FSH concentration required to elicit a maximal FSH-induced growth response was 67 mIU/mL [16]. Follicles were cultured at 37 °C in a humidified environment containing 5% CO_2_. Every other day, half of the culture medium was exchanged with fresh medium and stored at − 20 °C for hormone measurements. Culture was continued for 13 days.

To evaluate potential interactions between androgens and pioglitazone in early folliculogenesis, DHT (Tokyo Kaken, Tokyo, Japan) and pioglitazone (Tokyo Chemical Industry, Tokyo, Japan) were added to the culture media. Follicles from four mice (12 follicles/mouse/group) were randomly assigned to one of four culture conditions: 1) control (CTRL) group, base media plus DHT vehicle (100% ethanol) and pioglitazone vehicle (dimethyl sulfoxide; DMSO); 2) DHT group, CTRL media plus 500 ng/mL DHT and DMSO; 3) pioglitazone group, CTRL media plus 5 ng/mL pioglitazone and ethanol; and 4) DHT + pioglitazone group, CTRL media plus 500 ng/mL DHT and 5 ng/mL pioglitazone.

### Follicle survival and growth

Follicle survival and growth were assessed at days 1, 6, and 13 using an SMZ18 inverted microscope system (Nikon, Tokyo, Japan). Follicles were considered to be degenerating if oocytes became dark or were ejected outside of the follicle, if granulosa cells were dark and lysed, or if the diameter of the follicle decreased. The diameter of each follicle was determined by the averaging of two perpendicular measurements using NIS Elements Documentation D 3.22.00 (Nikon).

### Measurement of estradiol and vascular endothelial growth factor

Concentrations of estradiol (E2) and vascular endothelial growth factor (VEGF) in the culture media were measured at day 13 using an estradiol ELISA test kit (Neogen, Lansing, MI) with a detection range of 0–0.2 ng/mL, according to the manufacturer’s instructions. VEGF levels were measured using a VEGF ELISA test kit (R&D Systems, Abingdon, UK), with a detection range of 0–200 ng/mL, according to the manufacturer’s instructions. Interassay and intraassay coefficients of variance in these kits were below 10%.

### RNA extraction, reverse transcription, and real-time quantitative polymerase chain reaction

At day 13 of culture, five to ten follicles in each experimental group were analyzed for mRNA expression. Each follicle was ruptured using a 30-gauge needle, and the follicle wall and cumulus cells were collected for RNA extraction. Total RNA was isolated using an Absolutely RNA Nanoprep Kit (Agilent, Santa Clara, CA) according to the manufacturer’s instructions. Complementary DNA was synthesized from 1 μg of total RNA using a Superscript II Reverse Transcriptase kit (Thermo Fisher Scientific). Quantitative polymerase chain reaction (qPCR) was carried out using a TaqMan gene expression assay and AB StepOne Plus Real-Time PCR System (Foster City, CA). Gene expression levels of FSH receptor (*Fshr*) (Assay ID: Mm00442819_m1), androgen receptor (*Ar*) (Assay ID: Mm00442688_m1), phosphatase and tensin homolog (*Pten*) (Assay ID: Mm00477208_m1), anti-Müllerian hormone (AMH) (*Amh*) (Assay ID: Mm00431795_g1), AMH receptor 2 (*Amhr2*) (Assay ID: Mm00513847_m1), bone morphogenetic protein (BMP) 2 (*Bmp2*) (Assay ID: Mm0132882_m1), *Bmp6* (Assay ID: Mm 01332882_m1), activin A receptor type 1 (*Acvr1*) (Assay ID: Mm01331069_m1), BMP receptor type 1a (*Bmpr1a*) (Assay ID: Mm00477650_m1), BMP receptor type 1b (*Bmpr1b*) (Assay ID: Mm03023971_m1), *Vegfa* (Assay ID:Mm00437306_m1), and glyceraldehyde 3-phosphate dehydrogenase (*Gapdh*) (Assay ID: Mm99999915_g1) were analyzed by the 2^-ΔΔCt^ method. The amplification program included 40 cycles of denaturation at 95 °C for 15 s and 60 °C for 60 s. All reactions were run in duplicate.

### Data analysis

Data are presented as mean ± standard error of the mean (SEM). Statistical significance was determined using one-way analysis of variance (ANOVA) and Student-Newman-Keuls post-hoc analysis with SigmaPlot version 13.0 (Systat Software, San Jose, CA) for comparison of data amongst different treatment groups. Differences were considered significant at *P* < 0.05.

## Results

**Fig. 1:**
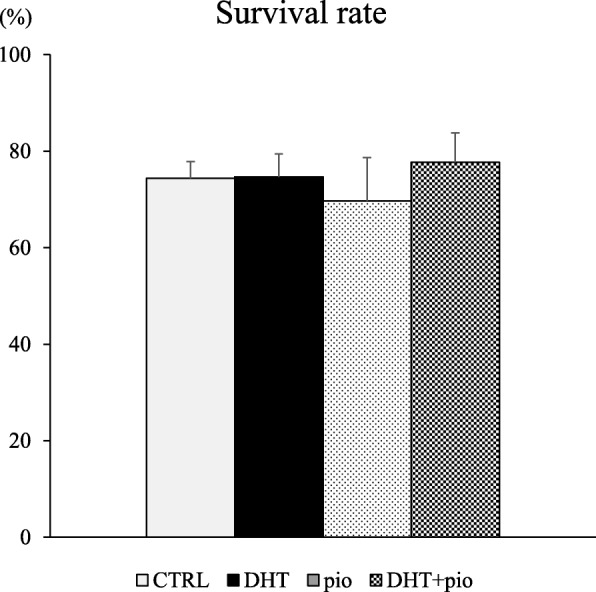
Effect of androgen (DHT) and insulin-sensitizer (pioglitazone) on follicle survival rates. Survival rates were determined under low follicle-stimulating hormone (FSH; 33 mIU/mL) at day 13 of culture. 1) Control (CTRL) group, base media plus DHT vehicle (100% ethanol) and pioglitazone vehicle (dimethyl sulfoxide; DMSO); 2) DHT group, CTRL media plus 500 ng/mL DHT and DMSO; 3) pioglitazone group, CTRL media plus 5 ng/mL pioglitazone and ethanol; and 4) DHT + pioglitazone group, CTRL media plus 500 ng/mL DHT and 5 ng/mL pioglitazone. Data are expressed as mean ± standard error. There was no significant difference in follicle survival rates among these groups (*P* = 0.833), by one-way ANOVA

**Fig. 2:**
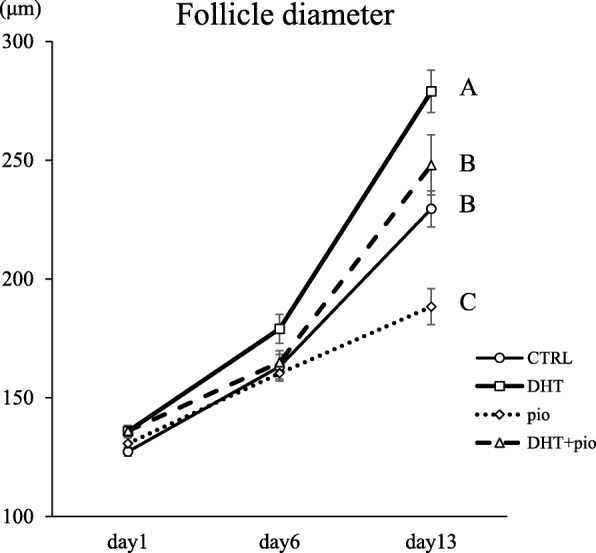
Effect of androgen (DHT) and insulin-sensitizer (pioglitazone) on average follicle diameters. Follicle growth was monitored at the indicated time points. 1) CTRL group, base media plus DHT vehicle (100% ethanol) and pioglitazone vehicle (dimethyl sulfoxide; DMSO); 2) DHT group, CTRL media plus 500 ng/mL DHT and DMSO; 3) pioglitazone group, CTRL media plus 5 ng/mL pioglitazone and ethanol; and 4) DHT + pioglitazone group, CTRL media plus 500 ng/mL DHT and 5 ng/mL pioglitazone. Data are expressed as mean ± standard error. Statistically significant differences among culture conditions are indicated by different letters

Upon evaluating follicle survival rates and diameters for the four experimental groups (Fig. 1), we found no significant differences in follicle survival rates among these groups (*P* = 0.833), or between the average diameters of surviving follicles at day 6 (*P* = 0.092) (Fig. 2). At day 13, the average diameter of follicles exposed to DHT (279.00 ± 8.96 μm) was significantly larger than observed in CTRL (229.51 ± 7.55 μm; *P* < 0.001) and DHT + pioglitazone (247.96 ± 12.64 μm; *P* = 0.020) groups. However, the average diameter of follicles in the pioglitazone group (188.31 ± 7.59 μm) was smaller than CTRL (*P* = 0.003) and DHT + pioglitazone (*P* < 0.001) groups. Moreover, the average diameter of follicles in the DHT group was larger than observed in the pioglitazone group (*P* < 0.001). Compared with the control group, the DHT group showed enhanced growth, while groups administered pioglitazone showed stagnation of the accelerated growth induced by DHT.

**Fig. 3:**
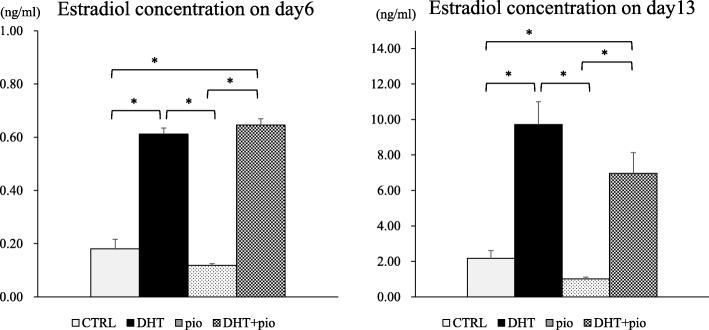
Estradiol (E2) concentrations in culture media at days 6 (panel **a**) and day 13 (panel **b**). 1) CTRL group, base media plus androgen (DHT) vehicle (100% ethanol) and pioglitazone vehicle (dimethyl sulfoxide; DMSO); 2) DHT group, CTRL media plus 500 ng/mL DHT and DMSO; 3) pioglitazone group, CTRL media plus 5 ng/mL pioglitazone and ethanol; and 4) DHT + pioglitazone group, CTRL media plus 500 ng/mL DHT and 5 ng/mL pioglitazone. Data are expressed mean ± standard errors. *P < 0.05, by one-way ANOVA and Student-Newman-Keuls post-hoc analysis

At day 6, E2 concentrations in the culture media were determined to be 0.18 ± 0.04 ng/mL in the CTRL group, 0.61 ± 0.02 ng/mL in the DHT group, 0.12 ± 0.01 ng/mL in the pioglitazone group, and 0.65 ± 0.02 ng/mL in the DHT + pioglitazone group (Fig. 3a). The E2 concentration in culture media of the DHT group was higher than observed in CTRL (*P* < 0.001) and pioglitazone groups (*P* < 0.001), while the concentration of E2 in culture media of the DHT + pioglitazone group was higher than observed in CTRL (*P* < 0.001) and pioglitazone groups (*P* < 0.001). E2 levels at day 13 exhibited a similar pattern as observed at day 6. E2 concentrations in the culture media were found to be 2.16 ± 0.45 ng/mL in the CTRL group, 9.72 ± 1.29 ng/mL in the DHT group, 1.02 ± 0.10 ng/mL in the pioglitazone group, and 6.97 ± 1.16 ng/mL in the DHT + pioglitazone group (Fig. 3b). The E2 concentration in culture media of the DHT group was higher than observed in CTRL (*P* < 0.001) and pioglitazone (*P* = 0.002) groups, and the E2 concentration in culture media of the DHT + pioglitazone group was higher than observed in CTRL (*P* = 0.03) and pioglitazone groups (*P* = 0.035).

**Fig. 4:**
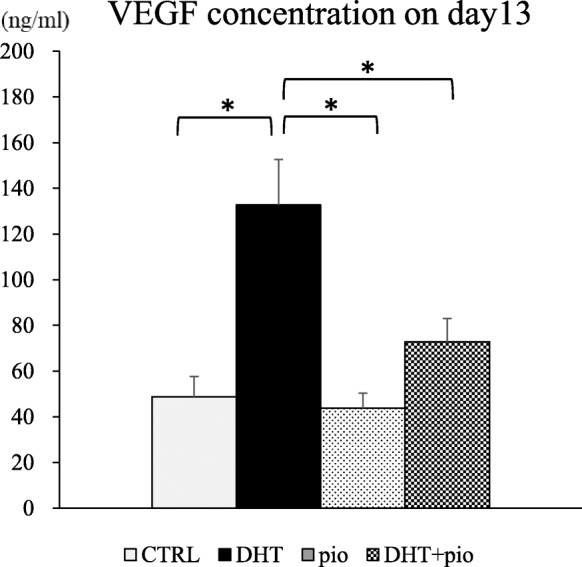
Vascular endothelial growth factor (VEGF) concentrations in culture media at day 13. 1) CTRL group, base media plus androgen (DHT) vehicle (100% ethanol) and pioglitazone vehicle (dimethyl sulfoxide; DMSO); 2) DHT group, CTRL media plus 500 ng/mL DHT and DMSO; 3) pioglitazone group, CTRL media plus 5 ng/mL pioglitazone and ethanol; and 4) DHT + pioglitazone group, CTRL media plus 500 ng/mL DHT and 5 ng/mL pioglitazone. Data are expressed as mean ± standard error. *P < 0.05, by one-way ANOVA and Student-Newman-Keuls post-hoc analysis

VEGF concentrations in culture media were found to be 48.79 ± 8.90 pg/mL in the CTRL group, 132.72 ± 19.94 pg/mL in the DHT group, 43.75 ± 6.50 pg/mL in the pioglitazone group, and 72.82 ± 10.17 pg/mL in the DHT + pioglitazone group (Fig. 4). The VEGF concentration in culture media of the DHT group was higher than that observed in CTRL (*P* < 0.001), pioglitazone (*P* = 0.036), and DHT + pioglitazone groups (*P* = 0.006). VEGF expression was significantly reduced when pioglitazone was added to culture media containing DHT.

**Fig. 5:**
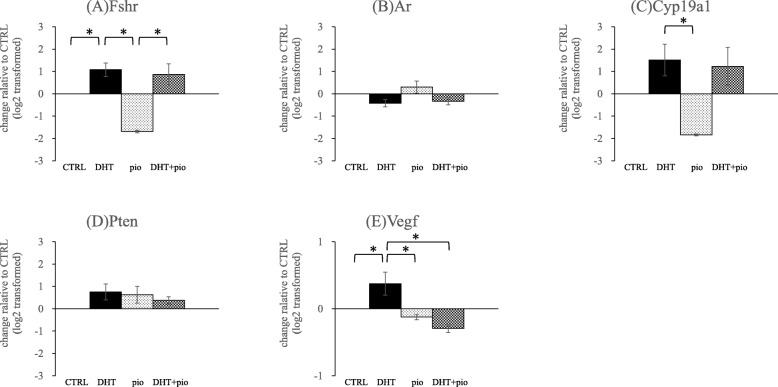
Effects of androgen (DHT) and insulin-sensitizer (pioglitazone) on gene expression of hormone receptors, *Cyp19a1*, *Pten*, and *Vegf*. Isolated follicles were cultured for 13 days and then analyzed for gene expression of **a** *Fshr*, **b** *Ar*, **c** *Cyp19a1*, **d** *Pten*, and **e** *Vegf*. 1) CTRL group, base media plus DHT vehicle (100% ethanol) and pioglitazone vehicle (dimethyl sulfoxide; DMSO); 2) DHT group, CTRL media plus 500 ng/mL DHT and DMSO; 3) pioglitazone group, CTRL media plus 5 ng/mL pioglitazone and ethanol; and 4) DHT + pioglitazone group, CTRL media plus 500 ng/mL DHT and 5 ng/mL pioglitazone. Data are expressed as mean ± standard error, and have been log_2_ transformed. All groups were compared with the control group. *P < 0.05, by one-way ANOVA and Student-Newman-Keuls post-hoc analysis

Exposure to DHT significantly increase *Fshr* mRNA expression (Fig. 5). Expression levels of *Fshr* mRNA in the DHT group were higher than that observed in CTRL (*P* = 0.040) and pioglitazone (*P* = 0.004) groups, respectively), and *Fshr* mRNA levels in the DHT + pioglitazone group were higher than that observed in the pioglitazone group (*P* = 0.007). C*yp19a1* mRNA levels in the DHT group were higher than that in the pioglitazone group (*P* = 0.029) (Fig. 5).

**Fig. 6:**
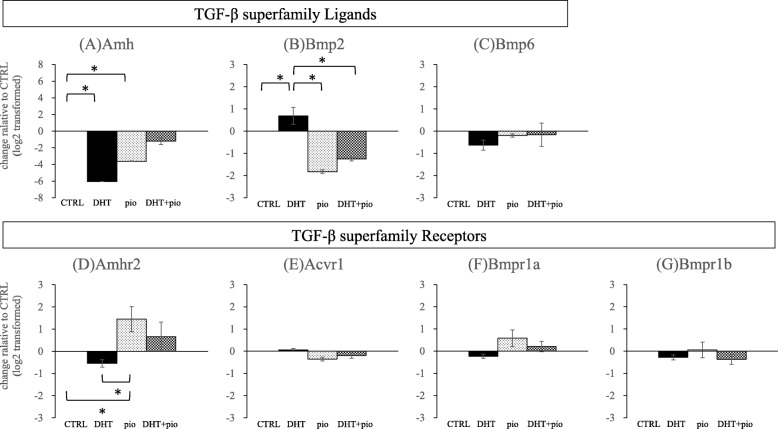
Effects of androgen (DHT) and insulin-sensitizer (pioglitazone) on gene expression of TGF-β superfamily ligands and receptors. Isolated follicles were cultured for 13 days and then analyzed for mRNA expression levels of **a** *Amh*, **b** *Bmp2*, **c** *Bmp6*, **d** *Amhr2*, **e** *Acvr1*, **f** *Bmpr1a*, and **g** *Bmpr1b*. 1) CTRL group, base media plus DHT vehicle (100% ethanol) and pioglitazone vehicle (dimethyl sulfoxide; DMSO); 2) DHT group, CTRL media plus 500 ng/mL DHT and DMSO; 3) pioglitazone group, CTRL media plus 5 ng/mL pioglitazone and ethanol; and 4) DHT + pioglitazone group, CTRL media plus 500 ng/mL DHT and 5 ng/mL pioglitazone. Data are expressed as mean ± standard error, and have been log_2_ transformed. All groups were compared with the control group. *P < 0.05, by one-way ANOVA and Student-Newman-Keuls post-hoc analysis

We also examined mRNA levels of several TGF-β superfamily ligands and receptors (Fig. 6). Expression levels of *Amh* mRNA in DHT and pioglitazone groups were lower than observed in the CTRL group (*P* = 0.027 and *P* = 0.024, respectively). Expression levels of *Amhr2* mRNA in the pioglitazone group were significantly higher than that observed in CTRL (*P* = 0.043) and DHT (*P* = 0.030). Expression levels of *Bmp2* mRNA in the DHT group were higher than that observed in CTRL (*P* = 0.050), pioglitazone (*P* = 0.002), and DHT + pioglitazone (*P* = 0.003) groups. Expression levels of *Vegf* mRNA in the DHT group were higher than that observed in CTRL (*P* = 0.045), pioglitazone (*P* = 0.036), and DHT + pioglitazone (*P* = 0.016) groups (Fig. 5). DHT treatment enhanced the expression of *Bmp2* mRNA, pioglitazone exposure suppressed induction of *Bmp2* mRNA by DHT. We did not detect any other significant differences in other genes analyzed amongst the different experimental groups.

## Discussion

On day 13 of culture, follicle survival rates for all experimental groups were between 70 and 80%, and showed no significant differences. Therefore, there were only minor technical problems in our follicle culture, and pioglitazone had no effect on secondary follicle survival. However, significant differences in follicular diameter were observed amongst the groups. As reported earlier, our present study also revealed that DHT significantly promoted secondary follicle growth by potentiating FSH action. In addition, follicle growth was suppressed by pioglitazone irrespective of the presence of DHT. Previous studies indicate that murine follicles acquire FSH-dependency once they reach a size between 160 μm and 180 μm in diameter [17–19]. Most follicles cultured in the presence of low FSH/pioglitazone could not grow to over 200 μm, indicating that growth stopped prior to the stage at which follicles become FSH dependent. That is, pioglitazone might affect secondary follicle growth by unknown pathways other than FSH signaling.

As previously reported, DHT administration increased the production of E2, which was associated with the promotion of follicle growth; thus, DHT administration increased both follicular growth and E2 production as a result of increased FHSR expression. Whereas, administration of pioglitazone tended to inhibit E2 overproduction by DHT, although it did not exhibit statistically significant differences. That is, effects of pioglitazone on secondary follicles might occur because of unknown pathways other than FSH signaling. To determine this mechanism, we investigated the gene expression profile involved in follicular growth using qPCR. qPCR results revealed a significant decrease in *Bmp2* transcription as a result of pioglitazone administration. With regard to transcriptional levels of *Bmp2*, the DHT group exhibited a significant increase compared with the control group; however, the addition of pioglitazone reduced this effect. Insulin sensitizers such as pioglitazone reportedly suppress the activity of the TGF-β superfamily [20]. Based on results of the present study, decreases in expression of BMP2 (a TGF-β superfamily member) might be associated with the effect of pioglitazone on suppression of follicle growth. BMP2 reportedly increases E2 production in the presence of FSH, but has little effect in the absence of FSH [21]. In this study, decreases in E2 elicited by pioglitazone were not prominent because of the low-FSH environment.

Other candidates potentially involved in the actions of pioglitazone actions are AMH and AMHR. Compared with the control group, the pioglitazone group exhibited decreased *Amh,* but significantly increased *Amhr*. Much remains to be determined about the action of AMH on follicle growth; however, it is thought to suppress follicle growth downstream of preantral follicles in mice [22]. The addition of pioglitazone leads to increased AMHR, which in turn may suppress the growth of preantral follicles by potentiating the action of AMH. Although AMH production in each follicle was decreased, the amount of AMH in vivo is still high enough because of a high number of preantral follicles within the PCOS ovary. This suggests that follicles underwent a sufficient amount of AMH action. Another AMH action is prevention of the transition from primordial to primary stages [22, 23]. In our experiments, growth was observed downstream of secondary follicles, therefore we were unable to determine the effect of pioglitazone on primordial follicles.

VEGF is known to increase during ovulation and OHSS [24]. In the present study, although the transcription of *Vegf* mRNA increased as a result of DHT, its transcription was suppressed by the addition of pioglitazone. ELISA analysis yielded similar results as qPCR with regard to protein levels. In addition, administration of insulin sensitizers has been reported to decrease serum VEGF levels [25], which is consistent with our results.

## Conclusions

Pioglitazone suppressed follicle growth and decreased VEGF levels, and may therefore have a preventative effect on OHSS. The results of the present study also suggest the involvement of BMP2 in the mechanism of follicular growth suppression. Thus, insulin sensitizers may improve the pathophysiology of PCOS patients and contribute to reducing the risk of severe complications associated with assisted reproductive technology.

## Data Availability

We would not share the data and material used in this manuscript, because we need them for further research.
